# Early dynamic changes in circulating tumor cells and prognostic relevance following interventional radiological treatments in patients with hepatocellular carcinoma

**DOI:** 10.1371/journal.pone.0246527

**Published:** 2021-02-12

**Authors:** Thomas J. Vogl, Linda J. Riegelbauer, Elsie Oppermann, Michel Kostantin, Hanns Ackermann, Annette Trzmiel, Stefan Stein, Katrin Eichler, Vladimir P. Zharov, Dhruvajyoti Roy, Andreas A. Schnitzbauer, Benjamin Strücker, Andreas Pascher, Wolf O. Bechstein, Mazen A. Juratli

**Affiliations:** 1 Institute of Diagnostic and Interventional Radiology, Frankfurt University Hospital, Goethe University, Frankfurt, Germany; 2 Department of General, Visceral and Transplant Surgery, Frankfurt University Hospital, Goethe University, Frankfurt, Germany; 3 Department of Biomedical Statistics, Frankfurt University Hospital, Goethe University, Frankfurt, Germany; 4 Flow Cytometry Unit, Georg-Speyer-Haus, Frankfurt, Germany; 5 Arkansas Nanomedicine Center, University of Arkansas for Medical Sciences, Little Rock, Arkansas, United States of America; 6 Laboratory for Advanced Medicine, Inc., Irvine, California, United States of America; 7 Department of General, Visceral and Transplant Surgery, Muenster University Hospital, Muenster, Germany; Humanitas Clinical and Research Center - IRRCS, ITALY

## Abstract

The aim of this study was to investigate the dynamic changes of circulating tumor cells (CTCs) in patients with hepatocellular carcinoma (HCC) before and immediately after conducting a microwave ablation (MWA) and conventional transarterial chemoembolization (C-TACE). Additionally, the CTCs short-term dynamics were compared with the clinical course of the HCC-patients. Blood samples from 17 patients with HCC who underwent MWA (n = 10) or C-TACE (n = 7) were analyzed. Venous blood was taken before and immediately after the radiological interventions to isolate and quantify CTCs using flow cytometry. CTCs were identified as CD45- and positive for the markers ASGPR, CD146 and CD274 (PD-L1). Patients were followed of up to 2.2 years after the radiological intervention. CTCs were detected in 13 HCC patients (76%) prior to the radiological interventions. The rate of CTCs was significantly decreased after the intervention in patients treated with MWA (0.4 CTCs/mL of blood, p = 0.031). However, no significant differences were observed in patients who received C-TACE (0.3 CTCs/mL of blood, p = 0.300). Overall, no correlation was found between the CTCs rate before and after the radiological intervention and recurrence rate of HCC. This preliminary data could confirm the tumoricidal effects of MWA in patients with HCC by significantly decreasing CTCs rate. In our study, we were able to detect CTCs in HCC patients using 3 different tumor markers. This preliminary data shows significant lower CTCs detected in response to MWA. However, large-scale randomized clinical trials are needed to determine the future role and the prognostic relevance of CTCs following this treatment.

## Introduction

Hepatocellular carcinoma (HCC), the most common subtype of primary liver cancer, is the third most frequent tumor-related cause of death worldwide [1]. Most frequently, intrahepatic tumor spread is observed in HCC. Common sites of extrahepatic tumor spread include the lung, abdominal lymph nodes and bones [2]. HCC is therefore generally a tumor with a high risk of recurrence [3].

Therapy options for the treatment of HCC include surgical procedures, chemotherapy, immunotherapy, and interventional radiological approaches [4]. After surgical liver resection, an increase in circulating tumor cell (CTC) count in venous blood of patients with cancer recurrence has already been reported [5]. For less invasive interventions such as microwave ablation (MWA) and conventional transarterial chemoembolization (C-TACE), the release of CTCs is also discussed as a potential complication [6,7]. In C-TACE, chemotherapeutic agents and embolization agents are applied directly into the hepatic vessels supplying the tumor via an arterial catheter [8]. The embolization agent leads to local ischemia and enables a longer effect and a higher concentration of the concomitantly applied chemotherapeutic agent in tumor tissue [8]. In MWA treatment, a microwave probe is placed into the tumor, which emits electromagnetic waves that destroy the tissue by generating heat [8].

In the last decade, CTCs have received a considerable amount of attention as potential diagnostic, recurrence and prognostic markers for tumors [9]. CTCs shed from primary and metastatic lesions through blood or lymphatic systems and they are easily accessible in peripheral blood [10]. Invasive diagnostic and therapeutic interventions for the tumor, such as biopsy or radiation, can lead to iatrogenic seeding of tumor cells [11–14]. Although CTCs have already been investigated in numerous clinical studies, their use in everyday clinical practice has not yet been established [9]. The detection of CTCs requires sensitive and specific analytical methods [14]. In addition, CTCs are rare in the peripheral blood of cancer patients and they must first be isolated from the other blood cells before they can be visualized e.g. by means of flow cytometry [15]. The detection of CTCs largely depends on the selection of surface markers and specificities of the corresponding fluorescence-labeled antibodies [14].

The aim of our study is to determine the dynamic changes of CTCs in patients with HCC before and immediately after MWA and C-TACE.

## Materials and methods

### Patient characteristics

In our prospective single-center study, 17 HCC patients, aged between 47 and 79 years (mean 64.6 ± 8.4 years, 12 male and 5 female, Table 1), were included. All patients received C-TACE or MWA as a therapeutic measure for the first time between September 2017 and June 2018. All procedeures were performed at the Institute of Diagnostic and Interventional Radiology, Frankfurt University Hospital. All patients were introduced in our interdisciplinary tumor boards before treatment. Seven patients were treated with C-TACE, and ten patients were treated with MWA. Peripheral blood samples from 13 healthy donors were collected in this study to determine cytometric settings and to determine the false positive rate. In all patients a high resolution MRI for the follow up was performed.

**Table 1:** Demographic characteristics of patients.

Baseline Characteristics	Patients, n = 17
**Gender (%)**	
Male	12 (70.6%)
Female	5 (29.4%)
**Age (years)**	
Mean ± SD	64.6 ± 8.4
Minimum	79
Maximum	47
**Etiology of liver disease**	
Alcohol	8 (47.1%)
NASH	3 (17.6%)
Autoimmune hepatitis	3 (17.6%)
Cryptogenic liver cirrhosis	3 (17.6%)
**BCLC staging**	
A	5
B	11
C	1
**Child-Pugh status (%)**	
A	9
B	5
C	3
**Recurrence within 2 years (%)**	
Yes	10 (58.8%)
No	7 (41.2%)

**Abbreviations**: SD, standard deviation; NASH, non-alcoholic steatohepatis; BCLC = Barcelona Clinic Liver Cancer staging.

### Study design

On the day of therapy, a total of 10 mL venous blood was taken from a peripheral venous catheter before the procedure and 10 minutes after the procedure commenced. The blood was stored in CellSave Preservative Tubes (Menarini Silicon Biosystems, Italy), which stabilizes the CTCs for up to 96 hours at room temperature. The blood in the CellSave Tubes was immediately processed within 24 hours, measured, and analyzed using FACSCAria Fusion, (BD Bioscience, Heildelberg, Germany). During every analysis of patient’s blood, a control blood sample from a healthy donor was also analyzed. However, if more than one procedure and analysis were performed on the same day, only one blood sample from a healthy donor was collected and analyzed. This study was approved by the ethics committee of the University of Frankfurt (approval number: 321/16). Informed consent forms were signed and obtained from all patients and healthy donors.

DOI of protocol: dx.doi.org/10.17504/protocols.io.bmyrk7v6.

### Cell line

HepG2 cells were purchased from CLS (Cell lines Service GmbH, Eppelheim, Germany) and cultured in DMEM/F-12 supplemented with 10% fetal bovine serum (FBS), 2% HEPES buffer, 1% GlutaMAX, and 1% Penicillin/Streptomycin (all: Gibco/Invitrogen). Cells were grown at 37 °C in a humidified incubator with 5% CO_2_. Authentication of HepG2 was performed by short tandem repeat (STR) genotyping (Cell lines service GmbH) and were mycoplasma free. Cells used in experiments were not more than 10 passages.

### Antibodies

Monoclonal antibodies (mAbs) already coupled with different fluorescent dyes were selected: Anti-Human Asialoglycoprotein Receptor 1 antibodiy (ASGPR-1)- R Phycoerythrin, clone 8D7 (BD Pharmingen, Heidelberg, Germany) to detect degenerated hepatocytes [16] Anti-Human CD146 antibodiy (also called Melanoma Cell Adhesion Molecule, MCAM)-APC, clone: SHM-57, Biolegend, San Diego, CA, USA to identify HCC cells. Recent studies have found CD146 to be overexpressed in the membranes of HCC cells, wheras healthy hepatocytes werde found to be negative for this marker [16]. The marker used for the aggressive CTCs was Anti- Human CD274-BV421, clone: MIH1 (BD Bioscience, Heidelberg, Germany) [17]. Anti-CD45-FITC, clone: HI30, Biolegend; San Diego, CA, USA, a known leukocyte antigen was used to detect the difference between tumor cells and leukocytes [18].

### Sensitivity and specificity of antibodies

To evaluate the sensitivity and specificity of our selected antibodies (Anti-ASGPR-1-PE, Anti-CD146-APC, Anti-CD274-BV421 and Anti-CD45-FITC), we used HepG2 cells and blood samples from healthy volunteers. Staining was optimized and repeated at least six times. (Table 2).

**Table 2:** Results of staining HepG2 cells healthy blood with the different antibodies followed by measurement using flow cytometry.

Antibody	Average of staining HepG2 cells alone with antibodies	Average of staining healthy blood alone with antibodies
ASGPR	98.96%	0.03%
CD146	99.10%	7.60%
PDL-1	2.30%	0.10%
CD45 (H130)	1.13%	98.30%

The number of tumor cells per staining was fixed at 1 x 10^5^ cells. The volume of blood used per staining was fixed at 1 mL. Each staining was performed a minimum of six times. The average result of the repetitions was calculated.

**Fig 1:**
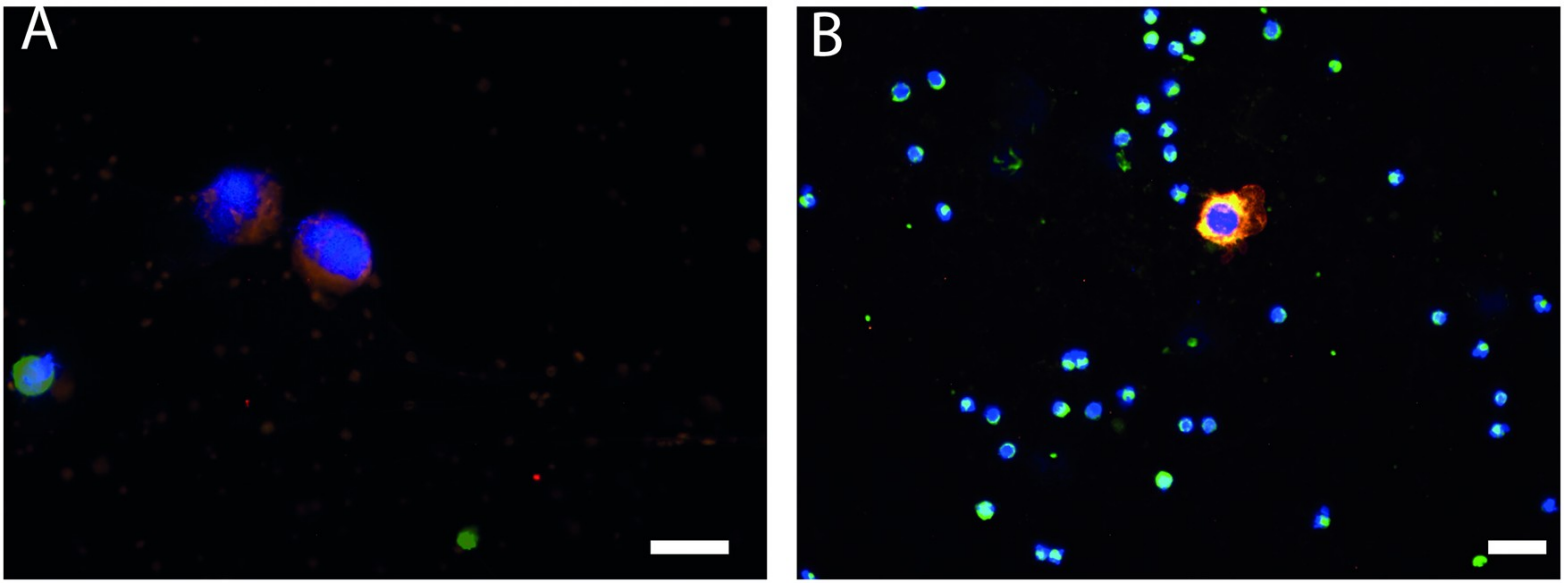
An exemplary picture of HepG2 cells exhibiting larger nuclei (DAPI) in tumor cells than in leukocytes and membrane staining of Anti-ASGPR-1-PE, Anti-CD146-APC, Anti-CD274-BV421 (merge). Leukocytes were stained with Anti-CD45-FITC. 40x magnification (A), 20x magnification (B). Scale 20 μm.

Furthermore, approximately 100 HepG2 cells were spiked into the blood of a healthy donor and CTCs were isolated using OncoQuick^®^ (Greiner Bio-One, Kremsmünster, Austria) and the staining, including 4’,6-diamidino-2-phenylindole (DAPI) (Southern Biotech, USA) for nucleated cells, was visualized under the immunofluorescence microscope (Zeiss Axio Observer Z-1, Zeiss, Jena, Germany, Fig 1).

### Enrichment and detection of CTCs

10 mL Blood from each patient and healthy volunteer were pipetted carefully above the filter of the pre-cooled OncoQuick^®^ tube according to manufacturer’s instruction and then centrifuged at 1600 x g, 4°C for 20 minutes. Subsequently, the isolated cells were washed twice with cold PBS without Ca++ and Mg++ containing 0.5% BSA (Bovine serum albumin, Sigma-Aldrich Chemie GmbH, Munich, Germany) and then transferred into FACS tubes. The enriched cells were stained, measured, and analyzed by FACSCanto II, BD Bioscience.

**Fig 2:**
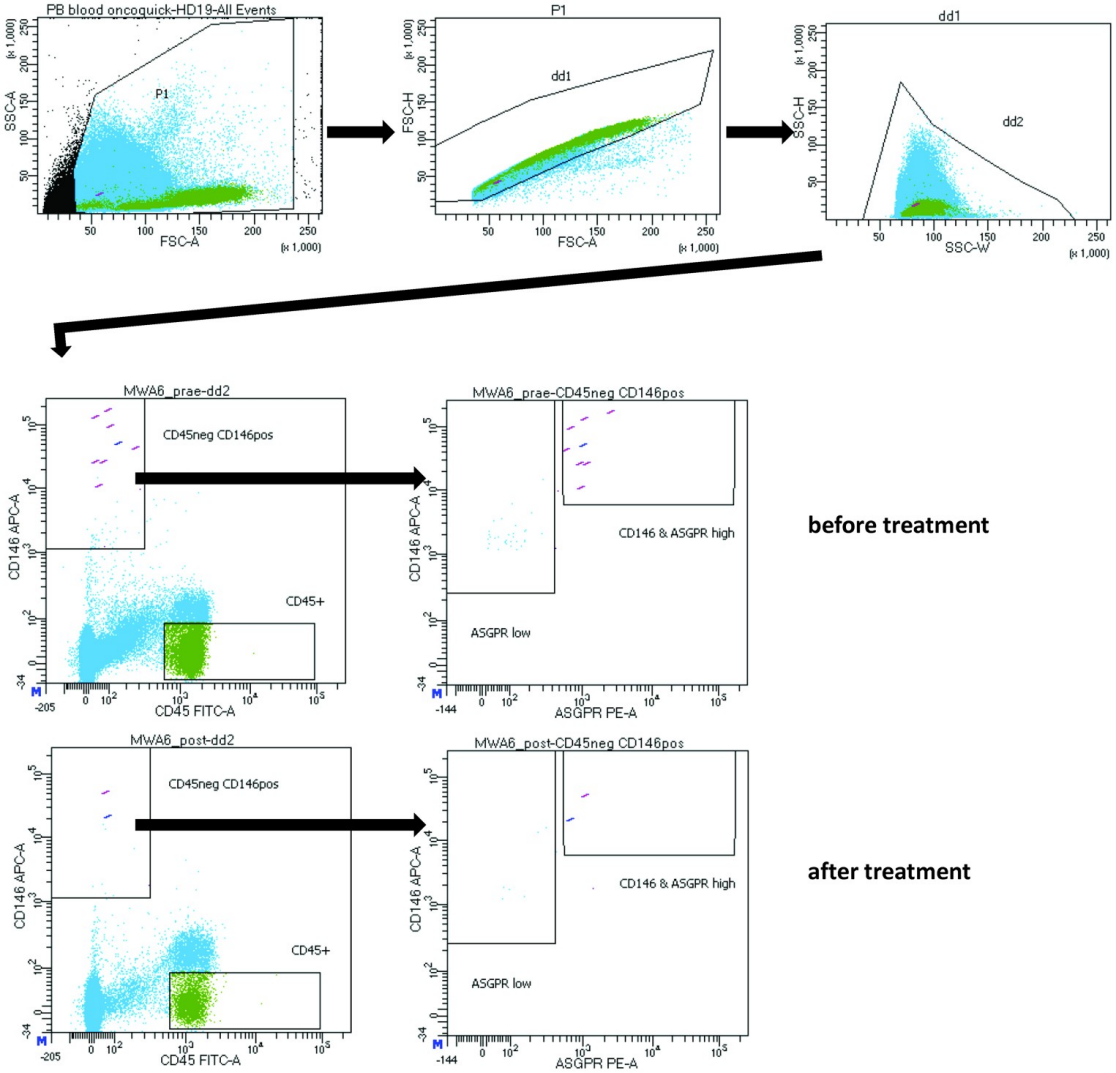
Flow cytometry gating strategy to identify the CTCs. FSC-A versus FSC-H and SSC-W versus SSC-H were used to eliminate cell doublets and other aggregates. Positive events (CD45neg, CD146^high^ & ASGPR^high^) were further gated for findings of CD274 (not shown).

A cocktail of fluorescent-labeled antibodies (Anti-ASGPR-1-PE, Anti-CD146-APC, Anti-CD274-BV421 and Anti-CD45-FITC) was added to the enriched CTCs in the FACS tubes immediately after tumor cell isolation, incubated for an hour at 4°C in the dark and washed twice with PBS containing 0.5% BSA. Finally, the CTCs were resuspended with FACS buffer, measured by four-laser FACSAria Fusion (BD Biosciences, CA, USA) and analyzed by FACS Diva Software (version BD FACSDiva 8.0.1). CTCs were defined as CD45-negative, and positive for antibodies against CD146, ASGPR-1, and CD274 (Fig 2).

### Microwave ablation technique

A microwave ablation device consists of three basic parts: generator, flexible cable and antenna (or the terms “applicator”, “probe”, “needle”). After desinfection, sterile covering of the access point and local anesthesia the puncture needle will be positioned under CT guidance and then it will be placed into the center of the tumor where it delivers thermal energy to destroy the tumor [19].

MWA makes use of electromagnetic energy, causing rotation of water molecules. Generally heat is dissipated centrifugally around the probe tip. When an adequate heat is generated throughout, tumor cells around the antenna tip can be destroyed effectively by denaturation of intracellular proteins and cell membranes through dissolution and melting of lipid bilayers. Intratumoral temperatures can be measured with a separately placed antenna. Intracellular proteins are denaturated and cell membranes are destroyed through dissolution and melting of lipid bilayers by heating the tissue above 45–55°C for about 5 minutes. At temperatures from 60°C-100°C immediate coagulation and at temperatures of more than 100–110°C vaporization and carbonization will occur [19]. The MWA was performed when the HCC-patient had up to 3 lesions ≤ 3 cm in diameter.

### Conventional transarterial chemoembolization technique

C-TACE is based on the effect of simultaneous application of chemotherapeutic drugs and embolic agents such as degradable starch microspheres (DSM), collagen and gelatine sponge (Gelofam), polyvinyl alcohol, or lipiodol. Commonly used chemotherapeutic agents are doxorubicin, epirubicin, mitomycin, cisplatin, and miriplanin [20].

After desinfection, sterile covering of the access point (inguinal region) and local anesthesia, a 5F-sheath is inserted into the femoral common artery using the Seldinger technique. After that, a 5F pigtail catheter is used for the aortography to gain an exploratory view of the abdominal arteries including the celiac trunk and the superior mesenteric artery. In a next step, selective catheterization of the celiac trunk using a 5F sidewinder catheter is performed. The angiography depicts the anatomy of the hepatic artery, tumor blush, feeding arteries, and arteriovenous shunts. In addition to the pre-interventional, contrast-enhanced CT or MRI, an indirect portography should be performed during angiography to ensure a stable flow in the portal vein. During the initial C-TACE, cone beam CT can be performed to evaluate the tumor-feeding artery and detection of small HCC lesions. A 2.8F coaxial microcatheter system is inserted through the celiac trunk and past the branches of the gastroduodenal artery. The microcatheter for the injection of chemotherapeutic drugs and embolic agents should be placed selectively or superselectively in the segment arteries which feed HCC lesions. After confirming the correct position of the catheter tip, the chemotherapeutic and embolic agents are infused under radiographic guidance. To control the correct administration of drugs and the occlusion of tumor vessels with flow stasis, a final angiography should be performed. After completing the procedure, the punctured location of the femoral artery must be occluded by using a percutaneous closure device or compression bandage. After the interventional treatment, patients should be transferred to an interventional ward for subsequent clinical observation. If no complications occur they can be discharged on the day of the procedure [20].

The C-TACE was performed in HCC-patients with multifocal disease with a diameter of ≤ 5 cm.

### Collected data and statistical analysis

Demographic data of the patients as well as laboratory and pathology findings, were taken from the electronic patient records. The data of the healthy volunteers were collected by verbal interview. The statistical evaluation was carried out with Prism 7 for Windows (Version 7.04) GraphPad Software, Inc, San Jose. A p-value < 0.05 was considered significant. CTCs were counted before treatment, and after treatment. Paired data sets were analyzed by Wilcoxon-matched-pairs signed rank test and Spearman r correlation. Following the examination of the patients’ blood, the data for monitoring the clinical course of the patients were collected in November 2019. Follow-up ranged from 1.4 and 2.2 years following the intervention.

## Results

### CTC analysis

Of all 17 HCC-patients, 13 (76.5%) patients were tested positive for CTC (Table 3). The average of detected CTCs before intervention was 1.29 CTCs/mL (0–8.7 CTCs/mL).

**Table 3:** Clinical course: Follow up to 2.2 years after treatment.

Patient	Etiology of liver disease	BCLC staging	Child-Pugh status	Clinical course	CTCs/mL before treatment	CTCs/mL after treatment
MWA 1	NASH	B	B	non- recurrence	0.6	0.5
MWA 2	NASH	C	A	Recurrence (local)	1.7	0.9
MWA 3	Alcohol	A	A	Recurrence (local)	3.7	1.9
MWA 4	Alcohol	B	A	Recurrence (local)	1.7	1.4
MWA 5	Alcohol	B	A	Recurrence (local)	0.3	0
MWA 6	Alcohol	B	B	Recurrence (local)	0.4	0.4
MWA 7	Alcohol	B	B	non- recurrence	0.0	0.0
MWA 8	Cryptogenic liver cirrhosis	A	C	Recurrence (local)	0.0	0.0
MWA 9	Cryptogenic liver cirrhosis	B	C	non- recurrence	8.7	8.1
MWA10	Alcohol	B	A	Recurrence (local)	0.0	0.0
C-TACE 1	Alcohol	A	A	non- recurrence	0.1	0.5
C-TACE 2	Autoimmune hepatitis	B	B	non- recurrence (died)	0.7	1.2
C-TACE 3	NASH	A	C	Recurrence (local + distant) (died)	0.9	0.3
C-TACE 4	Autoimmune hepatitis	B	A	Recurrence (local)	0.5	0
C-TACE 5	Autoimmune hepatitis	B	A	non- recurrence	0.6	0.5
C-TACE 6	Alcohol	B	B	Recurrence (local)	0.3	0.5
C-TACE 7	Cryptogenic liver cirrhosis	A	A	non- recurrence (died)	0.0	0.0

### The level of CTCs in blood according to clinical-pathological characteristics of the HCC-patients’ cohort

**Fig 3:**
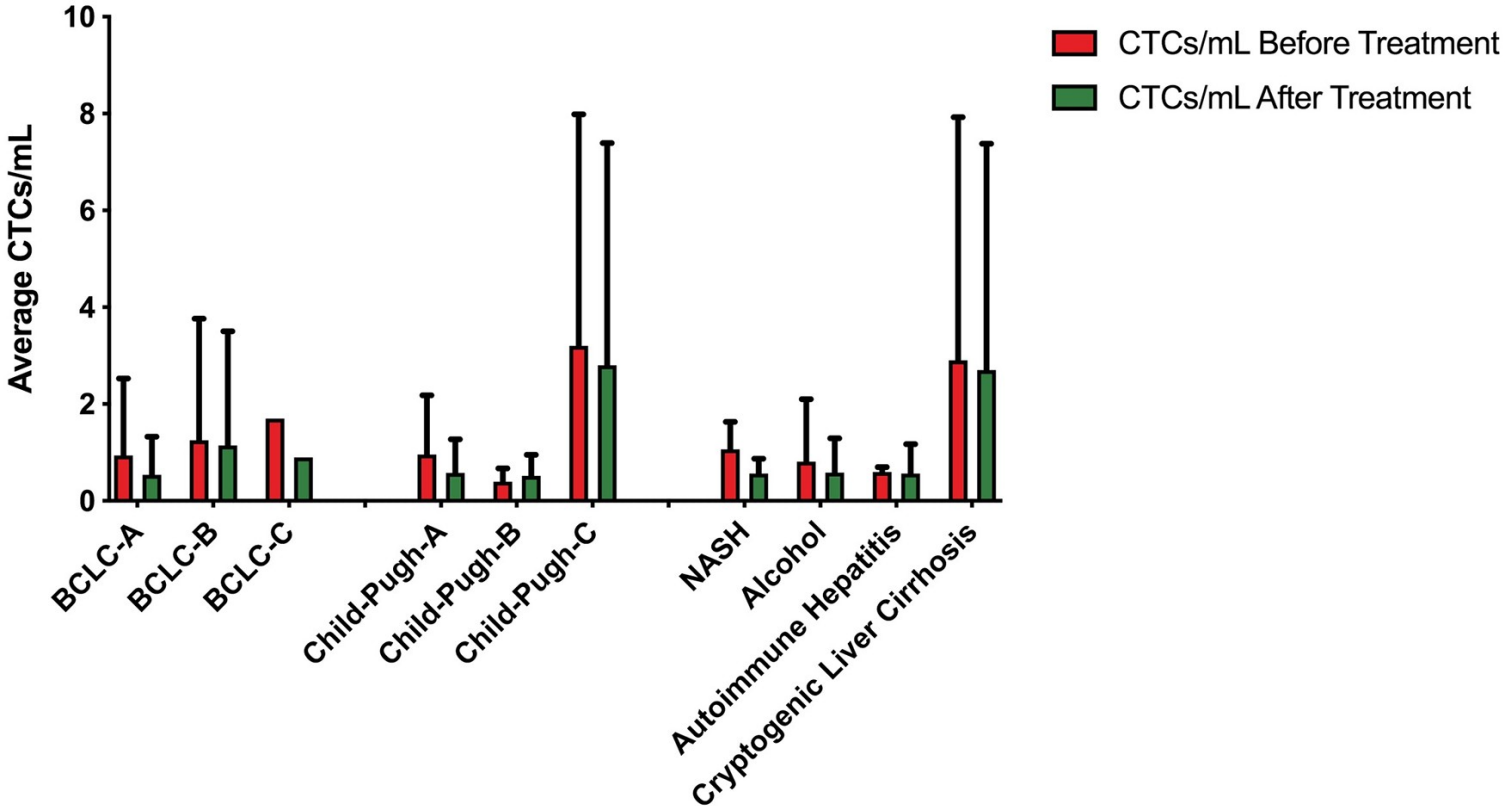
The level of CTCs per mL of blood before and after treatments (combined) according to clinical-pathological characteristics of the liver cancer patients’ cohort. Values and error bars represent the averages and SDs of CTC counts from patients with HCC. Significant, p <0.05.

The frequency of the average CTCs positivity is reported for different patient subgroups in relationship to BCLC tumor stages, patient’s Child-Pugh class, and liver disease etiology. The level of CTCs for different subgroups is summarized in Table 3. Patients were CTC positive in majority of all subgroups before treatment. However, at p ≥ 0.05 the comparison showed no significant (n.s.) difference for the tested subgroups (Fig 3).

### Short-term CTCs in MWA in HCC-patients

**Fig 4:**
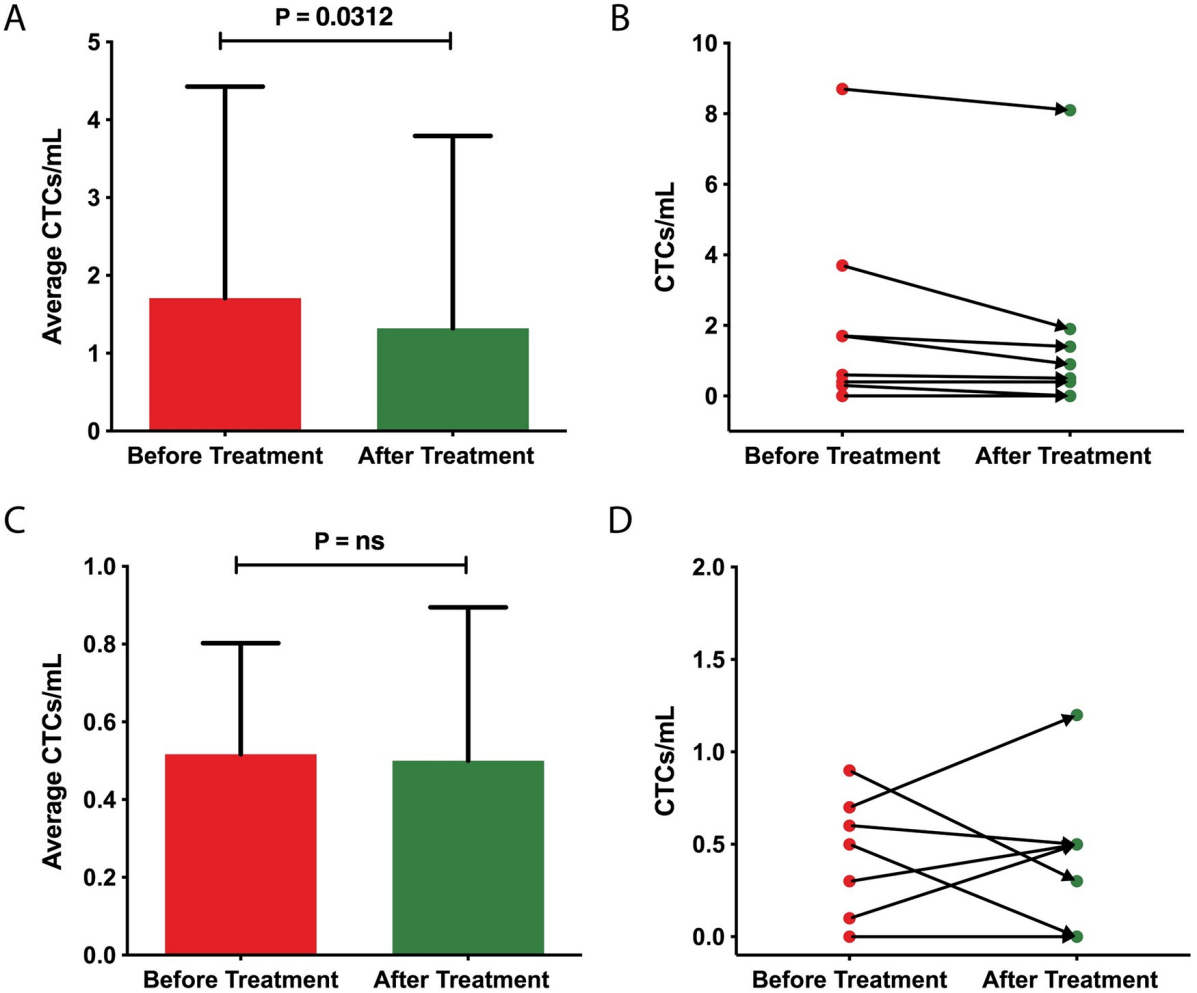
Early dynamic changes in CTCs following interventional radiological treatment in HCC-patients (A) Number of CTCs per mL of blood before and after MWA in n = 10 HCC-patients. Values and error bars represent the averages and SDs of CTC counts from n = 10 MWA-patients with HCC. Significant, p <0.05. (B) Short-term dynamic of CTCs before and after MWA in 10 HCC patients. In 3 patients who received MWA no CTCs were detected either before nor after treatment. (C) Number of CTCs per mL of blood before and after C-TACE in n = 7 HCC-patients. Values and error bars represent the averages and SDs of CTC counts from n = 7 C-TACE-patients with HCC. (D) Short-term dynamic of CTCs before and after C-TACE in 7 HCC patients. In one patient who received C-TACE no CTCs were detected neither before nor after treatment.

The mean CTC count of MWA patients (n = 10) before radiological intervention was 1.7 CTCs/mL of blood. After MWA was performed, the mean value was 1.3 CTCs/mL of blood. The comparison showed a significant decrease in the detected CTCs after the radiological intervention (n = 7, p = 0.031). In the 3 remaining patients no CTC was detected before and after treatment. The standard deviation was 2.7 CTCs/mL of blood for the samples before MWA and 2.5 CTCs/mL of blood for the samples after MWA (Fig 4A and 4B, Table 3).

### Short-term CTCs in C-TACE in HCC-patients

The mean CTC count of C-TACE patients with HCC (n = 7) before radiological intervention was 0.7 CTCs/mL of blood. After the C-TACE was performed, the mean value was 0.4 CTCs/mL of blood. After the treatment, in 3 patients the CTCs rate increased and in 3 patients decreased. In the 1 remaining patient no CTC was detected before and after treatment. Postinterventional the results were consequently lower by 0.3 CTCs/mL of blood (p = 0.3). The standard deviations were 0.6 CTCs/mL of blood for the samples before C-TACE and 0.4 CTCs/mL of blood for the samples after C-TACE (Fig 4C and 4D).

### Correlation between CTCs dynamic and therapy complete response

We found no significant correlation between mortality and the rate of CTCs before and after the MWA (n = 10) or C-TACE (n = 7) and recurrence rate or mortality in our HCC-patients (Spearman r = 0, p = ns). However, a complete response (CR) and no evidence of recurrence was observed in two patients. Patients were considered with CR if they showed tumor regression or no tumor progression, no local recurrence and no distant metastases. One patient from the group of MWA patients (10%) and one C-TACE patient (12.5%) were therefore classified with CR. Patients marked “recurrence” were alive but showed either tumor progression or local intrahepatic recurrence. This was present in seven of the MWA patients (70%) and in two C-TACE patients (28.6%). The clinical courses of the patients are shown in Table 3.

**Fig 5:**
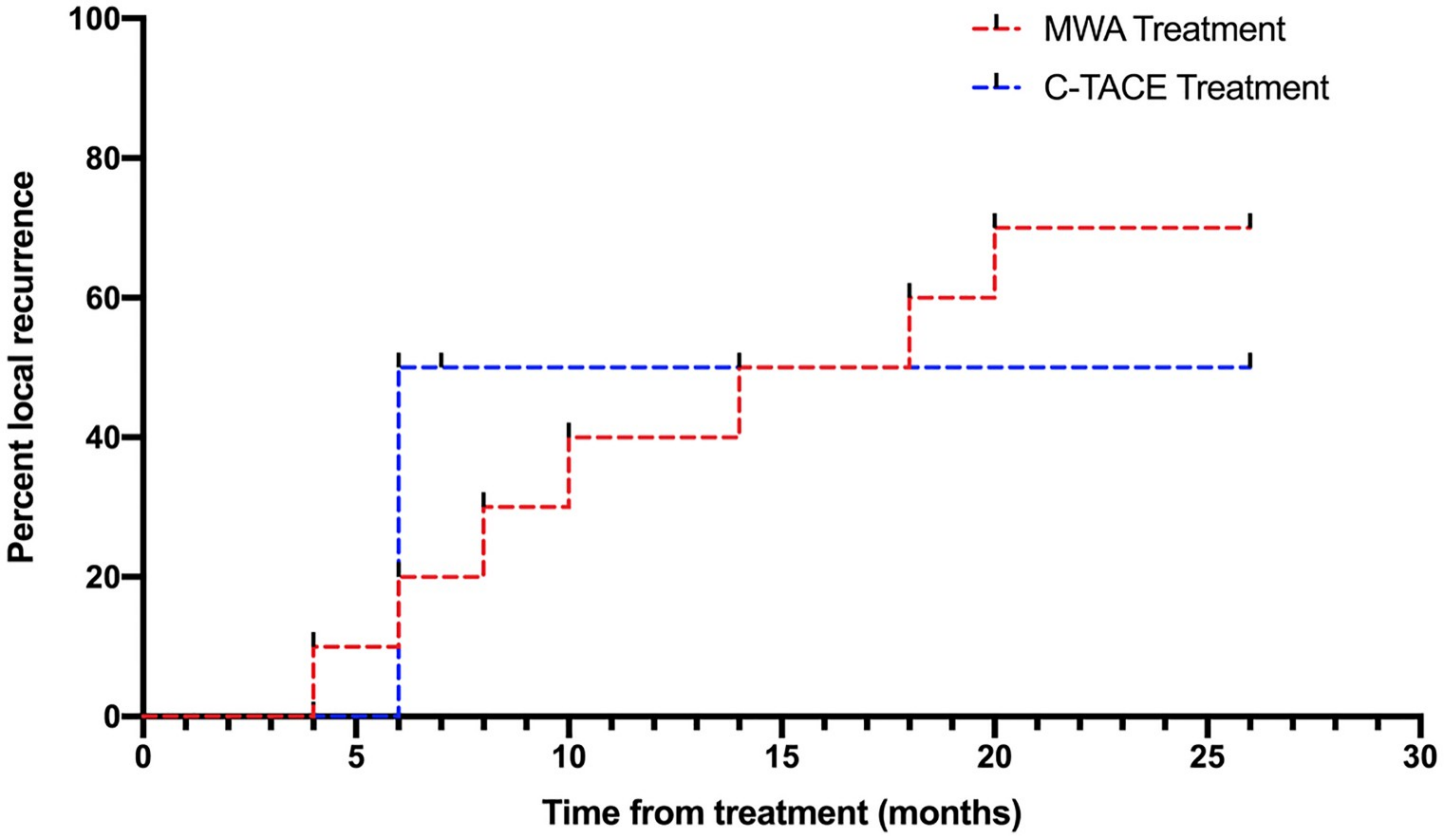
Kaplan-Meier analysis of time to first local recurrence following the treatments by using MWA versus C-TACE.

The Kaplan-Meier analysis conducted over a 2.2-year period following the treatments demonstrated no significant difference (χ^2^ = 0.053, p = ns) between the two procedures (Fig 5). The local recurrence rate in our study was in the normal range [21,22]. In all patients, intrahepatic recurrence was frequently detected due to high resolution MRI in the follow up.

## Discussion

The aim of our study is to determine the dynamic changes of CTCs in patients with HCC before and immediately after MWA and C-TACE.

Efficient isolation of CTCs from peripheral blood is challenging due to their extreme rarity (~1 CTC in every 10^9^ total blood cells) and their high phenotypic heterogeneity (physical characteristic differences between cells from the same tumor) [23]. Further complicating the large scale clinical utilization of CTC separation is that devices must be adaptable for a number of detection platforms (PCR, FISH, immuno-staining, etc.) [23] usable in a clinical setting and most importantly have scalable production capabilities.

In our study, the period between the two blood analysis before and after the procedure from the same patient was under 60 minutes, hence similar false positive rate in both samples is expected. Moreover, for false positive cell detection, blood from healthy donors was constantly analyzed together with patient’s sample to serve as internal control to set criteria in cytometric techniques for CTC identification.

Recent published studies showed that tumor manipulation, e.g. tissue biopsy, pressure and heating could increase the CTC release in animal models [5,24]. However, our results show that the CTC counts in the MWA group decreased significantly after treatment but there was no significant difference between before and after the treatment as far as C-TACE group is concerned which is probably due to the limited number of patients.

In four patients no CTCs were detected either before nor after the radiological intervention. This could be justified by the limitations of the *ex vivo* CTCs detection using FACS [20]. Additionally, the released cell populations are very heterogeneous in terms of surface markers, which create difficulties for detecting them in patient blood and thereby in clinical interpreting their presence using currently available methods [25]. One possible reason for non-CTC detection in some patients is the dynamic fluctuation of CTC rate in the blood vessels. These temporal CTC fluctuations can explain false negative results of a one-time snapshot test in humans [14].

Since C-TACE is more likely to be performed in more advanced disease, C-TACE patients could have been expected to have a higher CTC count in their blood than MWA patients. However, in our study, the average of detected CTCs before treatment was lower than in MWA. One reason could be a more advanced necrosis in C-TACE patients, accompanied by reduced blood flow to the liver and thus less CTCs in the blood before and after therapy. As discussed above, a possible reason for non-CTC or lower CTC detection in some patients is the dynamic fluctuation of the CTC rate in the blood vessels.

The greater drop in the CTC count in MWA compared with that in C-TACE could be the capacity to counteract potential tumor cell release by irradiating the puncture channel in MWA with microwaves when the probe is withdrawn to obliterate damaged vessels and destroy tumor cells carried along with the probe [6].

To our knowledge, this is the first study which determines the short-term effect of MWA on tumor cell release in patients with HCC. A previously published study with different methods of CTC isolation and detection showed a non-significant increase of CTCs in HCC patients after C-TACE in peripheral blood [26]. The study provided medians of CTCs of 0.13 CTCs pre- and 0.46 post-interventional per mL of blood [26]. The increase was explained by a malignant phenotype such as epithelial-mesenchymal transition (EMT) due to hypoxia after C-TACE [27]. In the present study, dedifferentiated cells and especially the aggressive types that are likely to undergo EMT were captured by using CD274. The most commonly used antigen in CTC detection is the epithelial cell adhesion molecule (Ep-CAM) because its expression in cells of epithelial origin is well investigated and absent in blood cells [28]. However, highly metastatic potential tumor cells could be missed because during the EMT process, the expression of epithelial markers is decreased [28]. The study on C-TACE and MWA presented in this paper did not use Ep-CAM but is based on CTC detection using an antibodiy cocktail of three different antibodies Anti-ASGPR, Anti-CD146 and Anti-CD274.

HCC metastasizes mainly through hematogenic spread [16]. Furthermore, it is known that vascular tumor invasion increases the risk of extrahepatic spread [29]. Therefore, a lower number of CTCs may be associated with a lower risk of metastasis or recurrence [30]. If the decrease in tumor cell count in the blood after a radiological intervention could be shown to be significant, this may indicate that the discussed theoretical risks of tumor cell release in C-TACE and MWA are perhaps nonexistent. In contrast, the benefit of C-TACE and MWA would not only be a direct attempt to eliminate the local tumor in the liver but also to reduce the CTC count in the blood of the patients and thus lower the risk of metastasis or recurrence after therapy [31].

One of the limitations of our study is the difficulties in detecting and analyzing CTCs include intertumor cellular heterogeneity and the rarity of CTCs. Even antibody cocktails targeting a wide variety of antigens may not account for the heterogeneity of CTC antigens [32]. An additional limitation of the human study is the small number of included patients.

## Conclusions

CTC detection could be an important diagnostic tool and possibly even enable new and more accurate classifications of HCC [33]. To the best of our knowledge, this is the first study to concomitantly investigate the dynamics of CTCs in patients with HCC before and immediately after receiving MWA and C-TACE. In our study, we were able to detect CTCs in HCC patients using 3 different tumor markers. This preliminary data shows significant lower CTCs detected in response to MWA. However, to make a statement to predict a clinical course based on the CTC difference, hence, further studies with a larger patient population must be conducted.
